# Biomarker panel for early screening of trastuzumab -induced cardiotoxicity among breast cancer patients in west virginia

**DOI:** 10.3389/fphar.2022.953178

**Published:** 2022-08-12

**Authors:** Sneha S. Pillai, Duane G. Pereira, Gloria Bonsu, Hibba Chaudhry, Nitin Puri, Hari Vishal Lakhani, Maria Tria Tirona, Komal Sodhi, Ellen Thompson

**Affiliations:** ^1^ Departments of Surgery and Biomedical Sciences, Marshall University Joan C. Edwards School of Medicine, Huntington, WV, United States; ^2^ Department of Oncology, Edwards Comprehensive Cancer Center, Marshall University Joan C. Edwards School of Medicine, Huntington, WV, United States; ^3^ Division of Cardiology, Department of Internal Medicine, Marshall University Joan C. Edwards School of Medicine, Huntington, WV, United States

**Keywords:** Cardiotoxicity after chemotherapy, biomarkers, Cardiac dysfunction, microRNA, breast cancer

## Abstract

Cardiotoxicity is a well-known pathophysiological consequence in breast cancer patients receiving trastuzumab. Trastuzumab related cardiotoxicity typically results in an overall decline in cardiac function, primarily characterized by reduction in left ventricular ejection fraction (LVEF) and development of symptoms associated with heart failure. Current strategies for the monitoring of cardiac function, during trastuzumab therapy, includes serial echocardiography, which is cost ineffective as well as offers limited specificity, while offering limited potential in monitoring early onset of cardiotoxicity. However, biomarkers have been shown to be aberrant prior to any detectable functional or clinical deficit in cardiac function. Hence, this study aims to develop a panel of novel biomarkers and circulating miRNAs for the early screening of trastuzumab induced cardiotoxicity. Patients with clinical diagnosis of invasive ductal carcinoma were enrolled in the study, with blood specimen collected and echocardiography performed prior to trastuzumab therapy initiation at baseline, 3- and 6-months post trastuzumab therapy. Following 6-months of trastuzumab therapy, about 18% of the subjects developed cardiotoxicity, as defined by reduction in LVEF. Our results showed significant upregulation of biomarkers and circulating miRNAs, specific to cardiac injury and remodeling, at 3- and 6-months post trastuzumab therapy. These biomarkers and circulating miRNAs significantly correlated with the cardiac injury specific markers, troponin I and T. The findings in the present study demonstrates the translational applicability of the proposed biomarker panel in early preclinical diagnosis of trastuzumab induced cardiotoxicity, further allowing management of cardiac function decline and improved health outcomes for breast cancer patients.

## Introduction

The manifestation of cardiotoxicity induced by chemotherapeutic agents is a well-established pathophysiological consequence which may lead to chronic, progressive and often irreversible cardiac damage (Florescu et al., 2013). The mitigation of the cancerous growth using conventional course of treatment by cytotoxic chemotherapeutic agents often presents with cardiovascular risks. Hence, it is essential to identify strategies to prevent chemotherapy related cardiac dysfunction (CRCD) and improve long term health outcomes for patients. This is particularly relevant for female population afflicted with breast cancer, which accounted for more than 2 million new cases worldwide in 2020, making it the most common form of cancer detected amongst women (Lukasiewicz et al., 2021). Given the rural and poor socioeconomic characteristics of West Virginia, the factors such as obesity, diabetes, and access to mammography screening will influence the poorer outcomes for women with breast cancer in West Virginia (Abraham et al., 2009). Hence the unusually high incidence of breast cancer has a strong inverse correlation with both annual income and educational achievement, which resulted in ranking West Virginia as 41^st^ in the United States (Vona-Davis et al., 2008). Furthermore, approximately 1 in 4 women in West Virginia have been afflicted with breast cancer, according to the West Virginia Department of Health and Human Resources (WV DHHR). While treatment options for breast cancer varies depending on the differentiated subtypes, trastuzumab remains one of the common therapeutic regimens, a humanized monoclonal antibody engineered to specifically target human epidermal growth factor receptor 2 (HER2) proteins (Kitani et al., 2019; Waks and Winer, 2019)

Although administering trastuzumab to early and metastatic HER2+ breast cancer patients along with other chemotherapeutic treatments improves their survival by 50%, patients often develop early cardiomyopathy which can later progress to ventricular dysfunction succeeding treatment completion (Portera et al., 2008). This is suggested to be partially attributed to the mechanism of action through which trastuzumab regulates HER2 proteins. More specifically, the cardiotoxic effects are proposed to be induced by trastuzumab interfering with HER2 function in cardiomyocytes thereby impeding their cardioprotective effects as well as the increased production of reactive oxygen species (ROS) (Mohan et al., 2017). The cumulative line of evidence suggests that trastuzumab induces type II reversible cardiotoxicity thereby suggesting that the risk of developing cardiac damage is dose-dependent and can typically be reversed through modulation of treatment (Onitilo et al., 2014). These cardiotoxic effects are exhibited through decreased left ventricular ejection fraction (LVEF) as well as heart failure in more severe cases (Huszno et al., 2013; Nowsheen et al., 2018). In fact, nearly 25% of HER2+ breast cancer patients experience a significant decline in asymptomatic LVEF and as many as 4.0% of patients experience symptomatic heart failure (Romond et al., 2012; Goldhirsch et al., 2013).

Several strategies have been proposed to reduce trastuzumab-induced cardiotoxicity, though none are studied in controlled clinical trials. These strategies include establishing stringent LVEF criteria for patient selection, monitoring cardiac function during therapy, discontinuing potentially cardiotoxic therapy when cardiotoxicity arises, and instituting heart failure medications early (Armenian et al., 2017). Current standards of monitoring CRCD suggests performing periodic echocardiography, which not only merely identifies cardiac damage once it has already developed, but also lacks the sensitivity and specificity required to serve as an effective prognostic tool that can be utilized for early screening (Jensen et al., 2002; Swain et al., 2003; Lakhani et al., 2018). Furthermore, given the poor cost-effectiveness of this conventional method of serial echocardiography, there have been limited implementation of such strategy, which poses economic burden, in a rural community like West Virginia. It is important to identify alternative and more cost-effective strategies allowing a prompt identification of drug-induced cardiotoxicity to prevent their aggravation.

In this study, we aim to create a panel of biomarkers and circulating miRNAs, to detect trastuzumab induced cardiotoxicity, prior to manifestation of clinical deficits in cardiac function. These biomarkers, including cardiac myosin light chain 1 (cMLC1), growth differentiation factor 15 (GDF-15) and placental growth factor (PIGF), have been shown to be aberrant prior to any detectable functional or clinical deficit in cardiac function. In addition, integration of miRNAs, including miR-34a, miR-21, miR-133, miR-1 and miR-30e, which are having significant role in cardiac function, will offer a superior prognostic modality. The prognostic approach using comprehensive assessment of panel of biomarkers is minimally invasive, highly cost effective and provides high specificity, proving to be a superior modality over conventionally utilized serial echocardiography (Mayeux, 2004; Cho, 2011). The effective utilization of this panel of biomarker may allow early detection of cardiotoxicity, resulting in early implementation of treatment and/or chemotherapy cessation.

## Material and methods

### Study design and patient population

All studies were performed in accordance with the guidelines and regulations outlined in the Declaration of Helsinki on the use and enrollment of human research subjects. The study was approved by the institutional review board (IRB) and ethics committee of Cabell Huntington Hospital and Marshall University Joan C. Edwards School of Medicine, WV (IRB No.: 866164). Trained hospital personnel reviewed the electronic medical records (EMR) and ensured an appropriate selection of the eligible patients, with rigorous confidentiality measures and HIPAA compliance. All subjects voluntarily participating in the study were briefed about the use of blood sample for this clinical study, signed the informed consent form (ICF) and agreed to the study follow up protocols.

Specifically, a total of 17 Caucasian female subjects were recruited for the study, who were visiting the Edwards Comprehensive Cancer Center at Cabell Huntington Hospital, WV. All patients of age >18 years and <80 years having a new clinical diagnosis of invasive ductal carcinoma, Stages IA (T1b-1c) to III A, having positive HER-2 receptor status (by IHC or FISH), scheduled to receive anti-HER2 therapy consisting of trastuzumab (with or without pertuzumab) were included in the study. The first 12 to 18 weeks of trastuzumab was given in combination with taxanes (weekly paclitaxel x 12 weeks or docetaxel (with or without carboplatin) every 3 weeks x 6 cycles). The trastuzumab regimen was administered at 4 mg/kg intravenous (IV) loading dose on week 1 followed by 2 mg/kg IV weekly in combination with weekly paclitaxel starting week 1 for a total of 12 doses followed by maintenance dose of trastuzumab at 6 mg/kg IV q3 weeks to complete one year of treatment ; or 8 mg/kg IV loading dose on day 1 followed by 6 mg/kg q3 weeks in combination with docetaxel x 6 cyces followed by maintenance dose of trastuzumab at 6 mg/kg IV q3 weeks to complete one year in the adjuvant setting.

Blood specimens were collected, and periodic echocardiography was performed on patients consenting to the participation in the study, at predetermined intervals: prior to the initiation of trastuzumab therapy (baseline; T0), at 3-months (T1) and 6-months (T2) post-initiation of the trastuzumab therapy. According to the exclusion criteria, any patient <18 years or >80 years old, patients with any second cancer, concurrent or prior history of chemotherapy and/or chest radiation therapy, history of myocardial infarction, cardiomyopathy or any cardiovascular dysfunction, hereditary iron metabolism disorder and hyaluronan synthase 3 gene (HAS3) polymorphisms, hematologic disorder, autoimmune disease, or any chronic diseases were excluded from the study during the patient screening process, as described previously (Lakhani et al., 2021). In addition, patients with LVEF ≤50%, as determined by echocardiography, history of symptomatic or asymptomatic heart failure, use of antihypertensive medications, antibiotics, weight loss medications or use of any medication for a chronic disease were also excluded from the study during the review of EMR for patient eligibility (Lakhani et al., 2021). Based on the periodic echocardiography at the predetermined intervals, including baseline, 3-months and 6-months post trastuzumab therapy initiation, cardiotoxicity was determined in the subjects according to the guidelines set forth by Cardiac Review and Evaluation Committee: symptomatic heart failure with a reduction of ≥5% to <55% from baseline in LVEF or an asymptomatic heart failure with a reduction of ≥10% to <55% from baseline in LVEF (Sawaya et al., 2011; Sawaya et al., 2012; Florescu et al., 2013; Ky et al., 2014).

Another set of 17 female subjects were also enrolled which served as the healthy controls. These age and sex matched healthy controls were enrolled, having no new onset of invasive ductal carcinoma or prior clinical history of any form of cancer, cardiovascular disease, in addition to applicability of all the exclusion criteria defined above. The appropriate confidentiality measures and consenting protocols, using ICF, were followed as detailed above. Patients wishing to participate in the study consented to the withdrawal of blood specimen and echocardiography procedure.

### Collection of blood specimen

Blood specimen was collected from all the eligible patients meeting the inclusion/exclusion criteria, consenting to participate in the study, as described previously (Lakhani et al., 2018; Pillai et al., 2020; Lakhani et al., 2021). As defined in the study protocol, patient follow up was maintained and blood specimen was collected, by trained hospital personnel, at baseline (before initiation of trastuzumab therapy), at 3-months and 6-months post trastuzumab therapy initiation. At each study interval, approximately 10mL of blood was withdrawn from antecubital vein and collected in the BD Vacutainer tubes, as described previously (Lakhani et al., 2021). Within 30 min of collection, each blood specimen was processed by centrifugation at 4000 rpm for 10 min under temperature of 4°C. Plasma obtained from these samples was further aliquoted in appropriately labelled Eppendorf tubes to avoid continuous freeze-thaw cycles at the time of their use. All aliquots were stored at -80°C and utilized for the quantitative measurement of biomarker levels and assessment of circulating levels of miRNA expression.

### Quantitative assessment of plasma biomarkers

Plasma samples were used for the quantitative assessment of biomarkers by enzyme linked immunosorbent assays (ELISAs). Commercially available kits were used and the manufacturer’s protocol was followed for each of the following biomarkers: Human GDF-15 (Abcam, United States), Human PIGF (Abcam, United States), Human cardiac MLC1 (MyBioSource, United States), Human cardiac Troponin T (MyBioSource, United States) and Human cardiac Troponin I (Abcam, United States). Each assay was performed using technical duplicates for each sample to minimize statistical error. The manufacturer’s provided antigen-specific coated 96-well plate was used for each assay, and the color produced at the end of the assay was read at 450nm wavelength in BioTek ELx900 Absorbance Reader, as described previously. The concentrations for each biomarker in each sample was calculated based on the standard curve, and the resulting equation from the line of best fit.

### Expression of circulating plasma miRNA

Total RNA was extracted from human plasma samples using miRNeasy Serum/Plasma Kit (Qiagen, United States), according to the manufacturer’s protocol, followed by synthesis of cDNA using miRCURY LNA RT Kit (Qiagen, United States) with a total of 50 ng RNA for each reaction, as described previously. Next, miRNA specific primers were used, combined with SYBR Green master mix, to perform RT-PCR reaction of 7,500 Fast Real Time PCR System (Applied Biosystems, United States). The miRNA expression was normalized using an internal control and a synthetic spike-in. For every sample, two technical replicates were used for the qRT-PCR amplification, to minimize the statistical error. The averages of the comparative threshold cycle (C_t_) values were used to calculate the amplification and relative fold change expression in the final analysis. Following is the sequence of human miRNA primers (Qiagen, United States), used in the study:

hsa-miR-34a-5p -5′UGG​CAG​UGU​CUU​AGC​UGG​UUG​U

hsa-miR-21–5p -5′UAG​CUU​AUC​AGA​CUG​AUG​UUG​A

has-miR-133a-3p -5′UUU​GGU​CCC​CUU​CAA​CCA​GCU​G

hsa-miR-1-3p -5′UGG​AAU​GUA​AAG​AAG​UAU​GUA​U

has-miR-30e-5p -5′UGU​AAA​CAU​CCU​UGA​CUG​GAA​G

#### Transthoracic echocardiography assessment

Transthoracic echocardiography was performed in the Cardiology Clinic at Cabell Huntington Hospital, WV. Echocardiography was performed on all healthy controls as well as each subject with breast cancer, at predetermined intervals (baseline, 3-months and 6-months), as outlined above in the study protocol. 2D Doppler and color flow imaging was used by a certified echocardiography technician using Philip TE 33 with S transducer in an ICAEL-accredited laboratory, as described previously (Lakhani et al., 2018; Lakhani et al., 2021). Echocardiography procedures were performed in accordance with the guidelines set forth by the American Society of Echocardiography (Nagueh et al., 2016). Each echocardiography image was read by the physicians who were blinded to the study groups and subjects. The LVEF was further calculated by 2D imaging, as described previously (Shaver et al., 2016).

### Statistical Analysis

The study was designed, conducted, recorded, analyzed, and interpreted without any bias, further ensuring that all results and data generated are reproducible. The statistical analysis, for all the data for each biomarker and circulating miRNA expression, was performed using GraphPad Prism 8.0. Bartlett’s test was used for data for each biomarker at each study interval to ensure equal variance. The data was tested for normality and subjected to parametric analysis. To identify the statistical significance among the mean plasma levels of biomarkers and circulating miRNA expression, one-way ANOVA was performed, followed by Tukey’s post-hoc t-test for multiple comparison. All significance was assigned at p<0.05 or p<0.01 for confidence interval of 95% or 99%, respectively. Each bar represents values as means ± standard error or mean (SEM). Correlation analysis was performed between cardiac injury specific markers, cardiac troponin I and cardiac troponin T, and each biomarker and circulating miRNA expression. The extent of correlation was determined by Pearson’s *r* coefficient using a 95% confidence interval and choosing two-tailed p-value to determined significance (alpha = 0.05), as described previously (Pillai et al., 2020; Lakhani et al., 2021).

## Results

### Patient demographics, clinical profile and echocardiography assessment

The subjects with breast cancer had an average tumor size of 2.97 cm ± 0.53 (Table 1). The assessment of clinical parameters in the laboratory panel, showed no significant difference at any of the study intervals, baseline, 3- and 6-months (Table 1). These clinical parameters included albumin, alkaline phosphatase, alanine transaminase (ALT), aspartate aminotransferase (AST), total bilirubin, blood urea nitrogen (BUN), creatinine, total protein, and N-terminal (NT)-pro B-type natriuretic peptide (BNP). Furthermore, assessment of echocardiography parameters also showed no significant difference at any of the study intervals, suggestive of no apparent cardiac function decline in the overall population (Table 1). Since numerous clinical trials have defined cardiotoxicity as the serial decline in LVEF (Alexander et al., 1979; Schwartz et al., 1987; Seidman et al., 2002; Cocco et al., 2022), it is important to note that when each subject was assessed, there were 3 isolated events of cardiotoxicity noted. Specifically, these 3 subjects (approximately 18% of the total population) met the criteria of cardiotoxicity, defined by the Cardiac Review and Evaluation Committee, at 6-months post trastuzumab therapy showing a significant LVEF decline of 19% (LVEF of 42% at 6-months), 24% (LVEF of 50% at 6-month) and 6.6% (LVEF of 54% at 6-months) from baseline, developing either symptomatic or asymptomatic heart failure.

**TABLE 1:** Summary of patient demographics, clinical parameters, and echocardiography measurements. This table provides demographics of the study subjects including their clinical profile and echocardiography measurements at each study interval of baseline (prior to trastuzumab therapy initiation), 3-months and 6-months post trastuzumab therapy initiation. There was no statistical significance among any of the parameters at any defined study interval. Values are presented as means ± SEM.

	Healthy Controls	Invasive ductal Carcinoma	
		
Sample Size (n)	17	17	
Age (years)	61.2 ± 2.0	51.6 ± 3.2	
Tumor Size (cm)	N/A	2.97 ± 0.53	
Clinical Data (Patients with Invasive Ductal Carcinoma)			
	Baseline (Prior to trastuzumab therapy)	3-months (After trastuzumab therapy)	6-months (After trastuzumab therapy)
Albumin (g/dl)	3.7 ± 0.1	3.6 ± 0.1	3.3 ± 0.2
Alkaline Phosphatase (U/L)	85.8 ± 6.6	82.0 ± 5.7	82.7 ± 6.8
SGPT (ALT) (U/L)	25.4 ± 4.1	33.3 ± 9.2	28.4 ± 6.3
SGOT (AST)	17.6 ± 2.9	17.9 ± 2.4	19.2 ± 2.6
Bilirubin, Total (mg/dl)	0.51 ± 0.09	0.36 ± 0.06	0.44 ± 0.08
BUN (mg/dl)	12.9 ± 1.4	11.7 ± 1.3	13.1 ± 1.1
Creatinine (mg/dl)	0.85 ± 0.03	0.80 ± 0.04	0.87 ± 0.05
Protein, Total (g/dl)	7.6 ± 0.1	7.4 ± 0.1	7.3 ± 0.1
NT-proBNP (pg/ml)	141.1 ± 42.4	179.6 ± 68.8	146.9 ± 89.3
Echocardiography (Patients with Invasive Ductal Carcinoma)			
	Baseline (Prior to trastuzumab therapy)	3-months (After trastuzumab therapy)	6-months (After trastuzumab therapy)
Systolic Blood Pressure (mmHg)	135.4 ± 3.6	131.9 ± 4.5	130.8 ± 3.8
Diastolic Blood Pressure (mmHg)	81.6 ± 1.4	80.4 ± 1.9	78.6 ± 2.5
Heart Rate (bpm)	78.6 ± 3.3	86.4 ± 2.2	85.1 ± 3.9
LV Ejection Fraction (%)	62.0 ± 1.3	58.4 ± 1.4	57.8 ± 2.0
LV Diastolic Volume (ml)	77.4 ± 3.9	78.5 ± 5.4	79.9 ± 5.0
LV Systolic Volume (ml)	29.8 ± 2.0	31.2 ± 2.9	33.9 ± 2.8
LV Stroke Volume (ml)	47.6 ± 2.5	47.3 ± 2.9	46.0 ± 3.3
LV Cardiac Output (L/min)	3.7 ± 0.2	4.1 ± 0.3	3.8 ± 0.3
LV Cardiac Index (L/min/m2)	1.9 ± 0.1	2.1 ± 0.1	1.9 ± 0.1

### Assessment of plasma biomarkers

**FIGURE 1:**
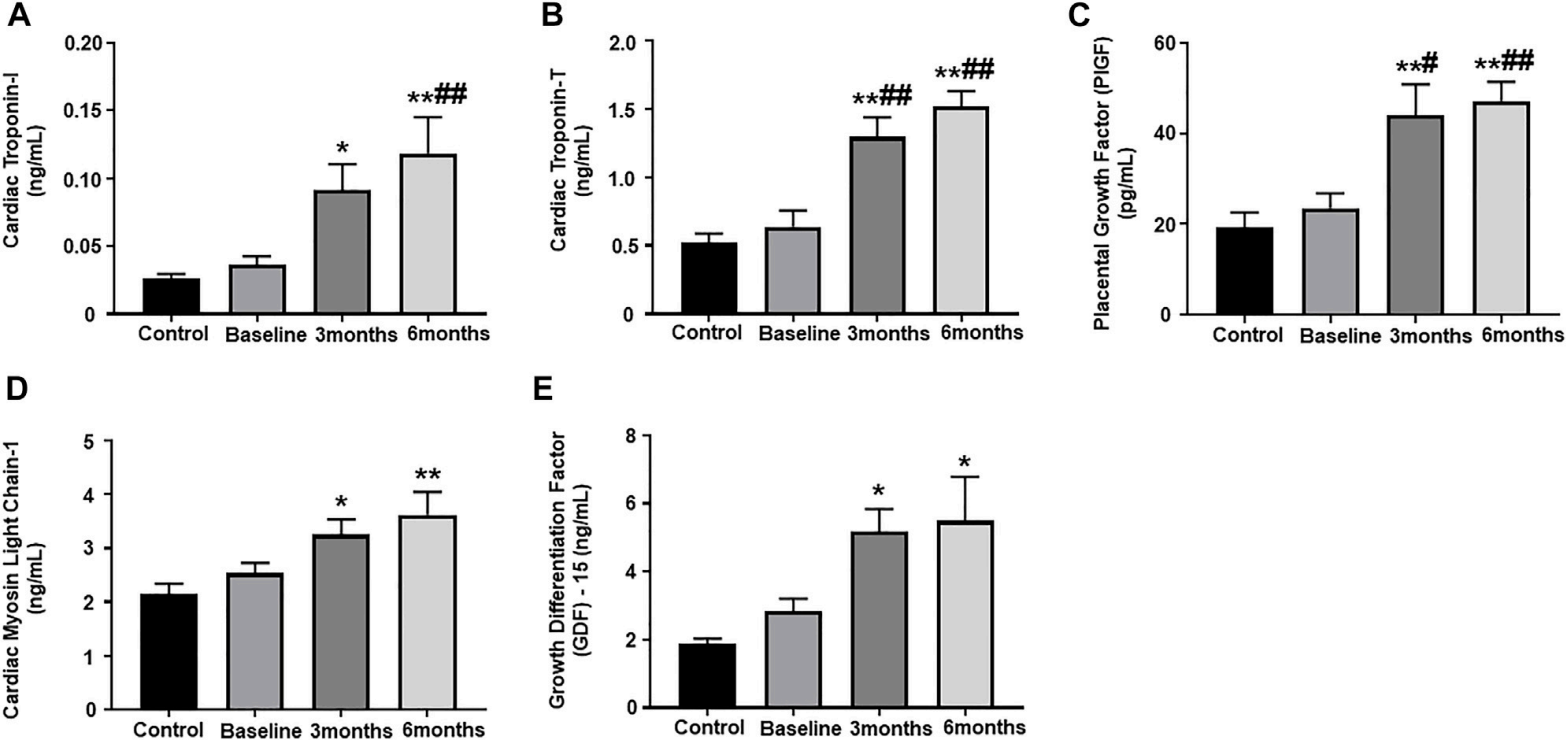
Assessment of plasma biomarkers. Quantitative analysis of plasma biomarker concentrations by ELISA: **(A)** cardiac troponin I, **(B)** cardiac troponin T, **(C)** placental growth factor (PIGF), **(D)** cardiac myosin light chain 1 (cMLC1), **(E)** growth differentiation factor 15 (GDF-15). Values represent means ± SEM. *p < 0.05 vs. Control, **p < 0.01 vs. Control, #*p* < 0.05 vs. Baseline, ##*p* < 0.01 vs. Baseline.

Our results showed significantly upregulated levels of cardiac troponin I and troponin T, which are highly specific markers of myocardial injury, at 3-months and 6-months post trastuzumab initiation, as compared to healthy controls (Figures 1A,B). When compared to baseline, the levels of cardiac troponin I was significantly upregulated at 6-months, while level of cardiac troponin T was significantly upregulated at 3- and 6-months (Figures 1A,B). Furthermore, we observed a significant upregulation of a pro-angiogenic marker, PIGF, at 3-month and 6-months post trastuzumab initiation, as compared to healthy controls and baseline (Figure 1C). Subsequently, the level of cMLC1, a marker of cardiomyocyte damage and injury, was also significantly upregulated at 3-months and 6-months, as compared to healthy controls (Figure 1D). Oxidative stress is one of the important mechanisms through which trastuzumab promotes cardiotoxicity, while previous studies have elucidated the functional role of GDF-15 in mediating oxidative stress. To this end, our study showed significantly elevated levels of GDF-15 at 3-and 6-months post-trastuzumab initiation, as compared to healthy controls (Figure 1E). There was no significant difference and progression in the levels of any of these biomarkers between 3-months and 6-months.

### Assessment of circulating plasma miRNA expression

**FIGURE 2:**
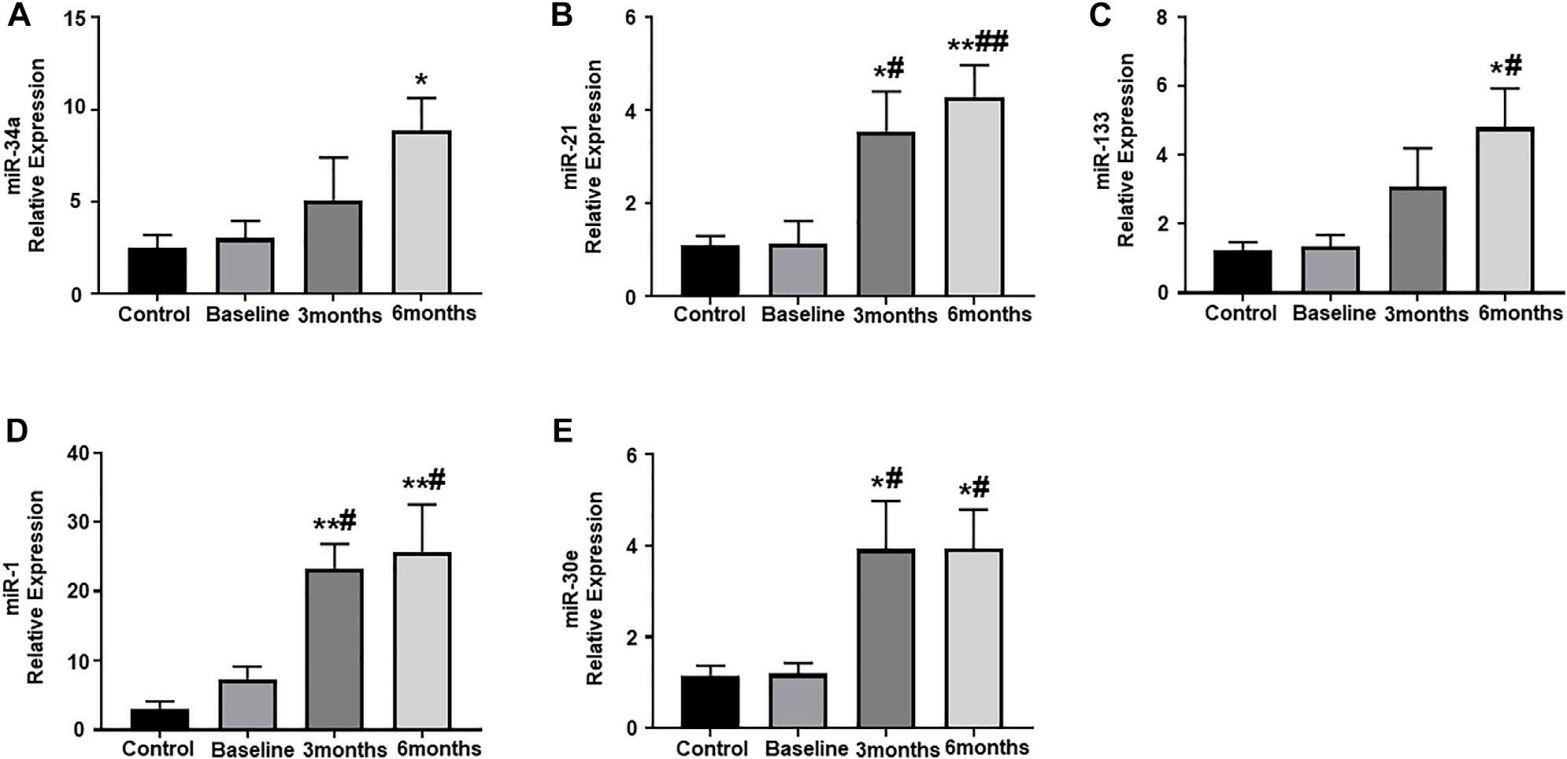
Assessment of circulating plasma miRNA expression. Quantitative analysis of circulating plasma miRNA expression by qRT-PCR: **(A)** miR-34a, **(B)** miR-21, **(C)** miR-133, **(D)** miR-1, **(E)** miR-30e. Values represent means ± SEM. *p < 0.05 vs. Control, **p < 0.01 vs. Control, #*p* < 0.05 vs. Baseline, ##*p* < 0.01 vs. Baseline.

Plasma samples from our study subjects, obtained at pre-determined study intervals, were assessed for the expression of some important circulating miRNAs, which have been shown to play a crucial role in cardiac remodeling and exacerbation of cardiac dysfunction. In the present study, the expression of circulating miR-34a was significantly upregulated at 6-months post trastuzumab therapy initiation, as compared to healthy controls (Figure 2A). Subsequently, there was also significant upregulation in the expression of miR-21 at 3-and 6-month post trastuzumab therapy, as compared to baseline and healthy control (Figure 2B). The relative expression of miR-133 also showed marked increase at 6-months, as compared to baseline and healthy controls (Figure 2C). Next, we assessed the expression of miR-1 and miR-30e, which showed significant upregulation at 3- and 6-months, as compared to baseline and healthy controls (Figures 2D,E). There was no significant difference and progression in the levels of any of these circulating miRNAs between 3-months and 6-months.

### Correlation of cardiac Troponin I and T with the plasma biomarkers and circulating miRNAs

**FIGURE 3:**
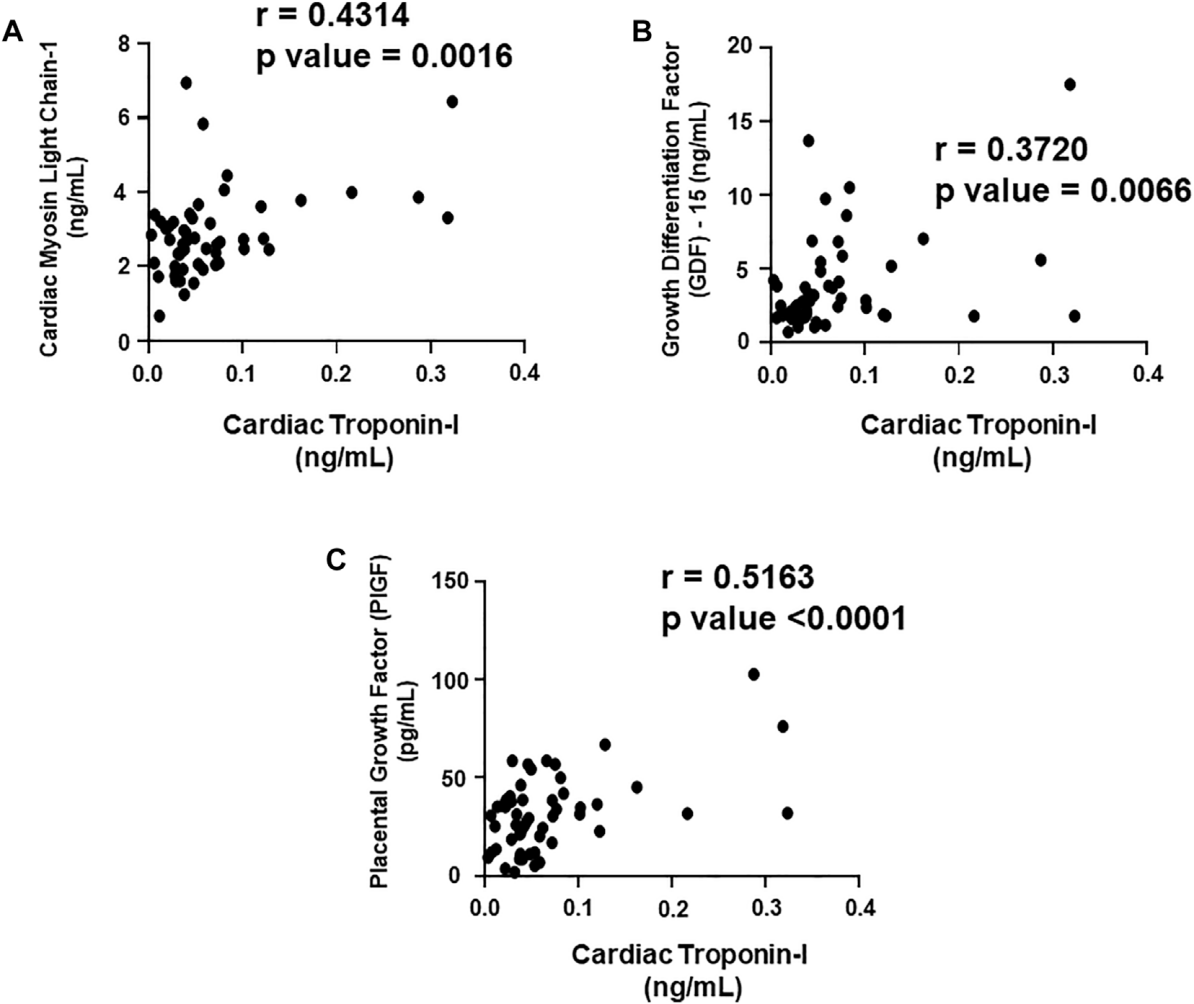
Correlation analysis of cardiac troponin I with plasma biomarkers. Correlation analysis was performed using a scatter plot between cardiac troponin I and plasma biomarkers, **(A)** cardiac myosin light chain 1 (cMLC1), **(B)** growth differentiation factor 15 (GDF-15) and **(C)** placental growth factor (PIGF). Pearson’s *r* coefficient was used to determine the extent of correlation and statistical significance was obtained by two-tailed *p*-value. Each plot independently demonstrates the correlation (Pearson’s *r* coefficient) and significance (*p*—value).

**FIGURE 4:**
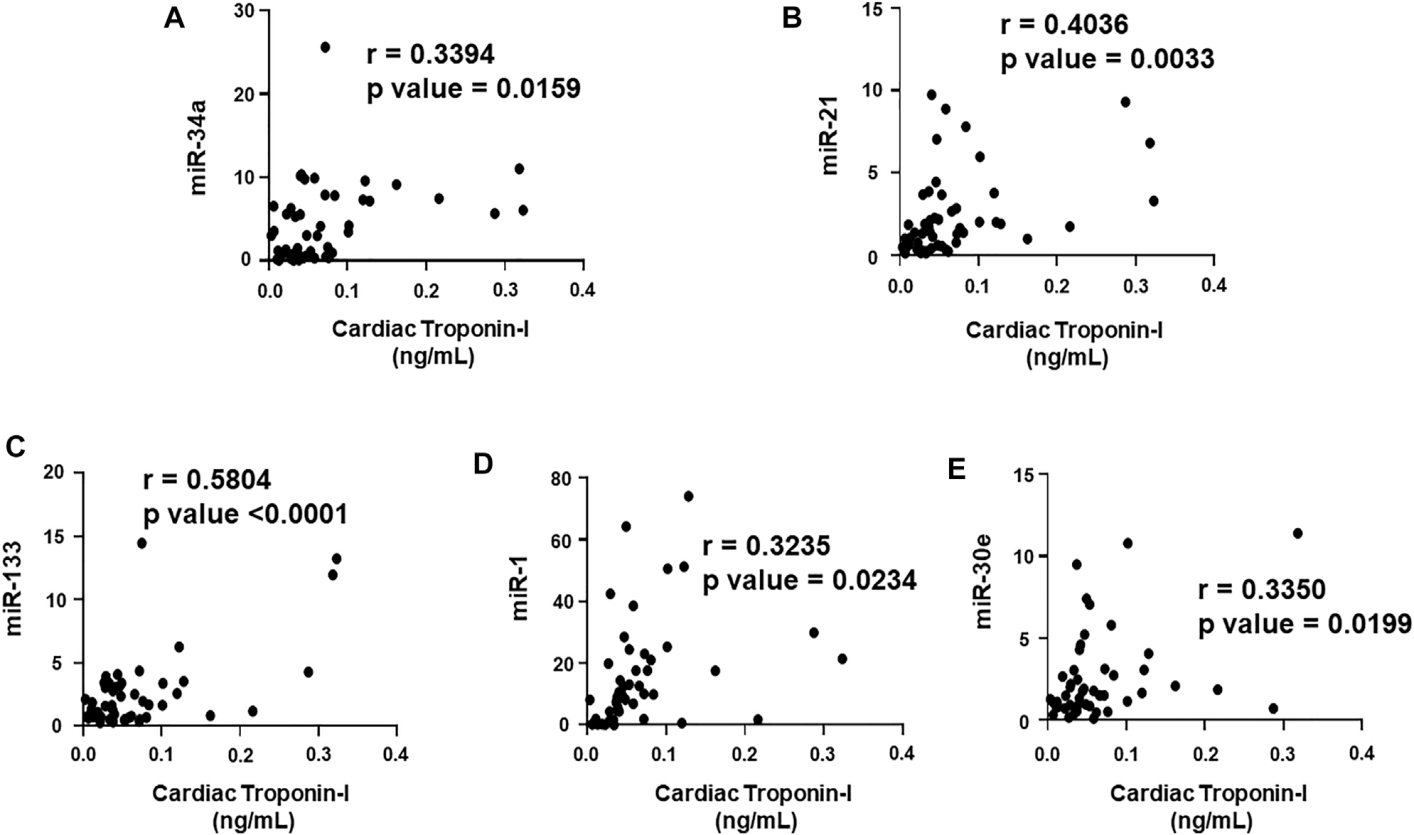
Correlation analysis of cardiac troponin I with circulating plasma miRNAs. Correlation analysis was performed using a scatter plot between cardiac troponin I and plasma miRNAs, **(A)** miR-34a, **(B)** miR-21, **(C)** miR-133, **(D)** miR-1, **(E)** miR-30e. Pearson’s *r* coefficient was used to determine the extent of correlation and statistical significance was obtained by two-tailed *p*-value. Each plot independently demonstrates the correlation (Pearson’s *r* coefficient) and significance (*p*—value).

**FIGURE 5:**
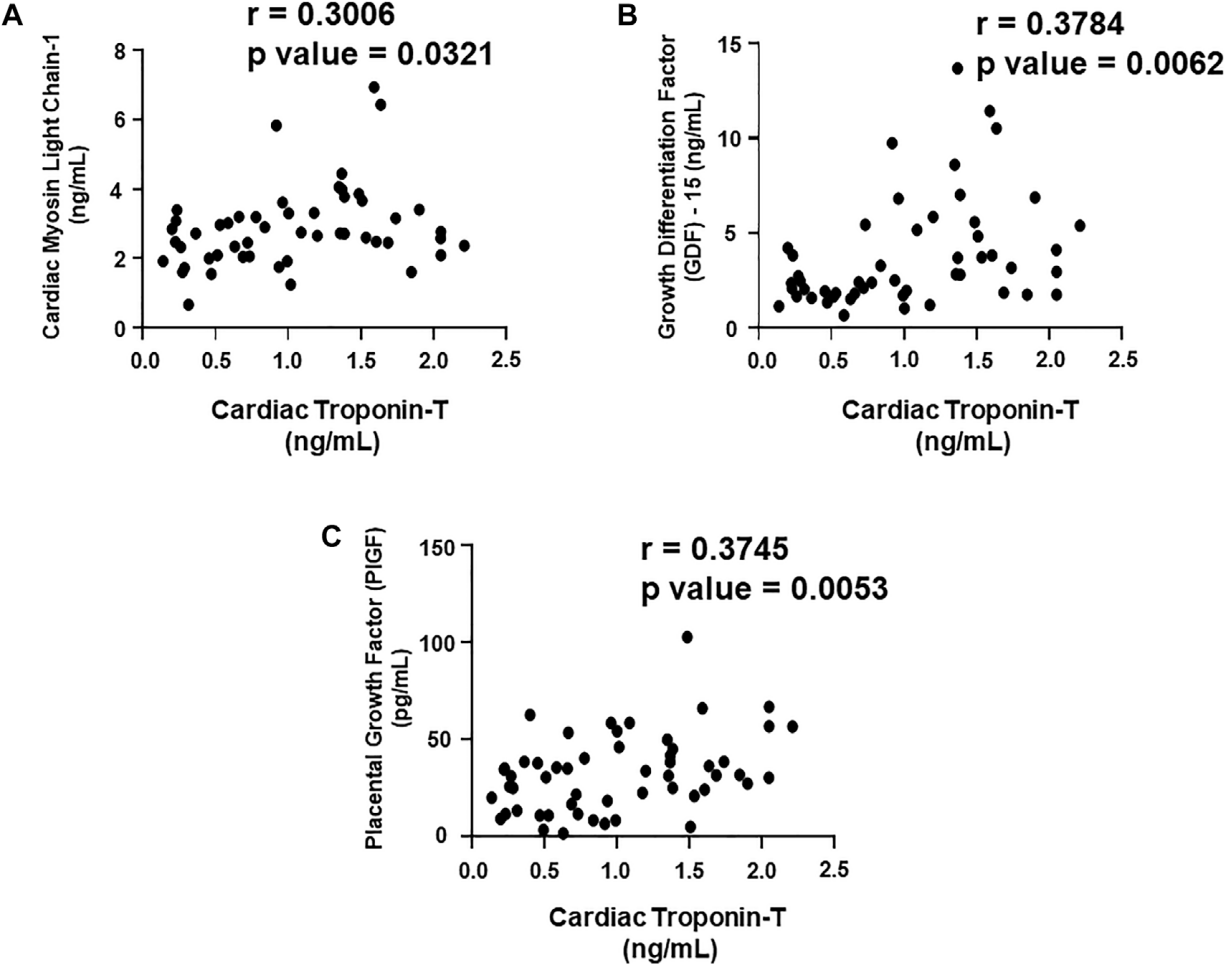
Correlation analysis of cardiac troponin T with plasma biomarkers. Correlation analysis was performed using a scatter plot between cardiac troponin T and plasma biomarkers, **(A)** cardiac myosin light chain 1 (cMLC1), **(B)** growth differentiation factor 15 (GDF-15) and **(C)** placental growth factor (PIGF). Pearson’s *r* coefficient was used to determine the extent of correlation and statistical significance was obtained by two-tailed *p*-value. Each plot independently demonstrates the correlation (Pearson’s *r* coefficient) and significance (*p*—value).

**FIGURE 6:**
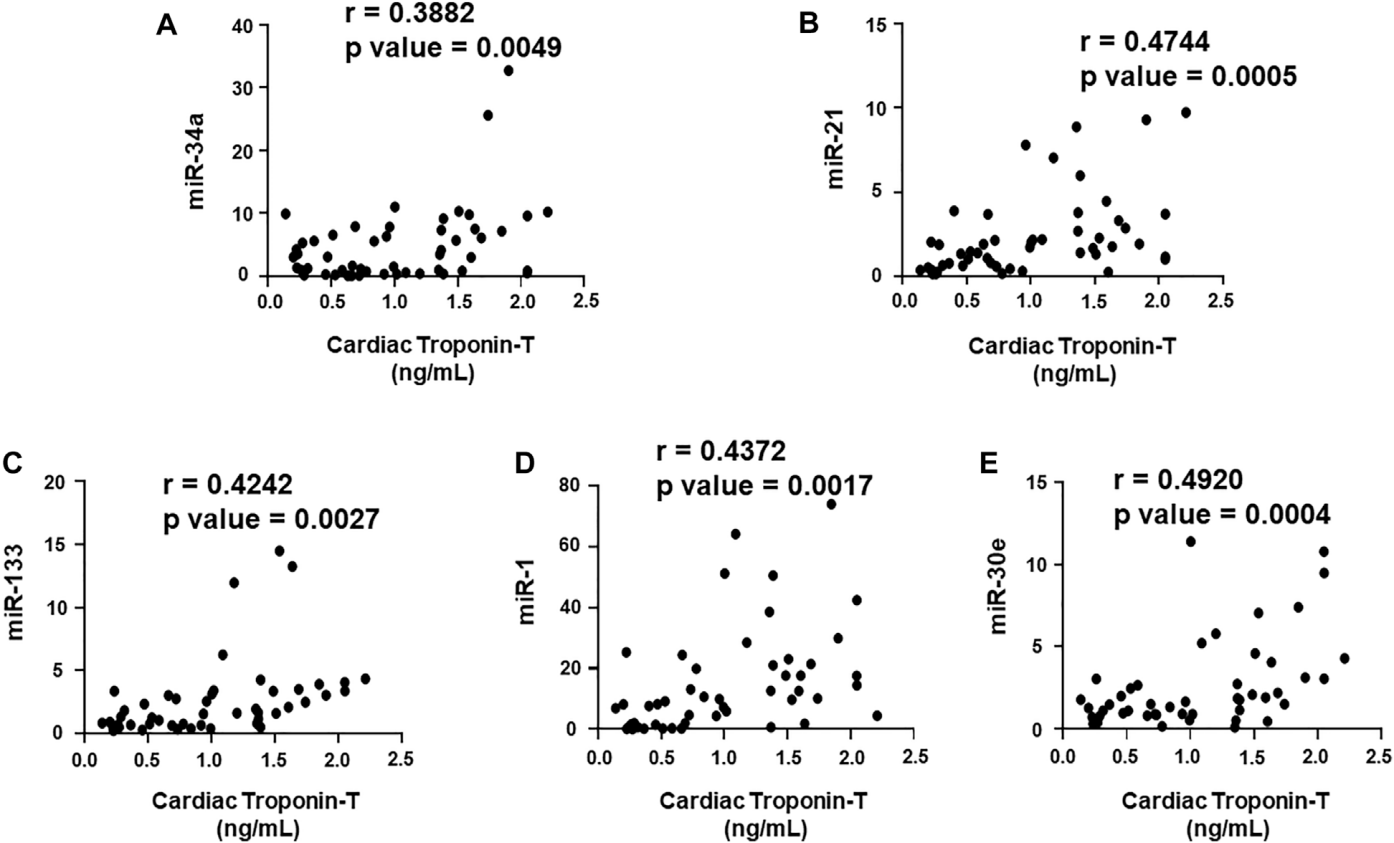
Correlation analysis of cardiac troponin T with circulating plasma miRNAs. Correlation analysis was performed using a scatter plot between cardiac troponin T and plasma miRNAs, **(A)** miR-34a, **(B)** miR-21, **(C)** miR-133, **(D)** miR-1, **(E)** miR-30e. Pearson’s *r* coefficient was used to determine the extent of correlation and statistical significance was obtained by two-tailed *p*-value. Each plot independently demonstrates the correlation (Pearson’s *r* coefficient) and significance (*p*—value).

We aimed to establish a correlation of our panel of biomarkers and miRNAs with cardiac Troponin I and T, which have been demonstrated to offer high sensitivity and specificity in response to changes in the left ventricular (LV) function. The extent of correlation was established by plotting each biomarker against either cardiac troponin I or troponin T and determining the Pearson’s *r* coefficient. Our results showed significant and positive correlation of cardiac troponin I with each biomarker, cMLC1 (r = 0.4314), GDF-15 (r = 0.3720) and PIGF (r = 0.5163), offering confidence interval of >99% (Figures 3A–C). Subsequently, cardiac troponin I also showed strong positive correlation with all miRNAs, miR-34a (r = 0.3394), miR-21 (r = 0.4036), miR-133 (r = 0.5804), miR-1 (r = 0.3235) and miR-30e (r = 0.3350) (Figures 4A–E). Furthermore, high sensitive cardiac troponin T also showed a significant and positive correlation with cMLC1 (r = 0.3006), GDF-15 (r = 0.3784) and PIGF (r = 0.3745) (Figures 5A–C). Finally, a significant positive correlation was observed between cardiac troponin T with each miRNA, miR-34a (r = 0.3882), miR-21 (r = 0.4744), miR-133 (r = 0.4242), miR-1 (r = 0.4372) and miR-30e (r = 0.4920), all at confidence interval of >99% (Figures 6A–E). Our correlation analysis offers strong evidence for the viability and utility of this panel of biomarkers for the early screening of trastuzumab induced cardiotoxicity in breast cancer patients.

## Discussion

**FIGURE 7:**
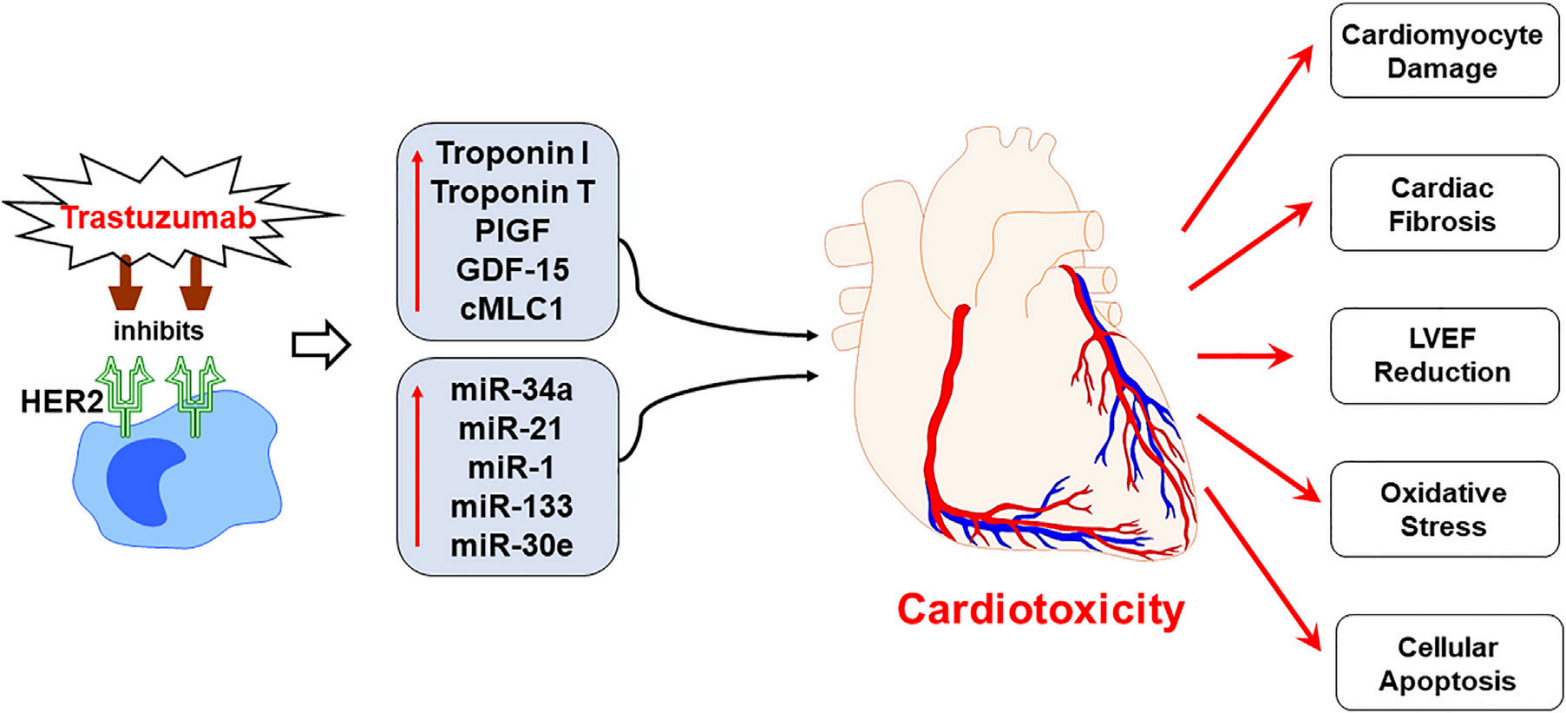
Schematic representation demonstrating the progression of trastuzumab induced cardiotoxicity. Chemotherapeutic regimen, trastuzumab, specifically inhibits the human epidermal growth factor receptor 2 (HER2) receptor to negate the cancerous cell growth and survival, as a mechanism of action. However, trastuzumab treatment upregulates the expression of plasma biomarkers and circulating miRNA, that are primarily involved in the regulation of cardiac function. The mechanistic action of trastuzumab induces cardiotoxicity by cardiomyocyte damage, cardiac fibrosis, LVEF decline, oxidative stress and cellular apoptosis. Since the panel of proposed biomarkers and miRNAs is specifically involved in the cardiac remodeling and regulation of cardiac function, their upregulated expression can predict cardiotoxicity before it is apparent on echocardiography. Hence, the utility of the proposed panel in clinical practice is viable for the early prognosis of trastuzumab induced cardiotoxicity.

Cardiotoxicity emerges as one of the most prevalent and a well-known pathophysiological consequence of chemotherapy in breast cancer patients. The chemotherapy induced cardiotoxicity is characterized by reduction in LVEF, which is often augmented by the progressive cardiac damage caused by the mechanisms specific to the chemotherapeutic regimens, including trastuzumab (Figure 7). Although the rates for new female breast cancer cases have been steadily increasing in the past four decades, the mortality have only slightly decreased, which is attributed to the development of effective multimodality treatments. Cardiac comorbidity present high risk for the patient receiving adjuvant and neoadjuvant therapies consisting of trastuzumab (Onitilo et al., 2014), thereby hindering the potential benefits posed by these chemotherapeutic treatments by significantly reducing the quality of life for survivors. However, trastuzumab-induced cardiotoxicity is dose-dependent (Copeland-Halperin et al., 2019), indicating that appropriate cardiac surveillance during chemotherapy may enable prevention as well as attenuation of intrinsic cardiac damage prior to its onset. The current standard of detection is by serial echocardiography, a non-invasive procedure that is conducted every three to six months (Tan and Scherrer-Crosbie, 2014). Although this method is effective at detecting cardiotoxicity, it lacks the specificity required to for the prognosis of progressive cardiac degeneration before it manifests into detectable cardiac dysfunction (Sawaya et al., 2012). Global longitudinal strain (GLS) is also another technique being used in clinical practice in order to detect early changes in left ventricular myocardial contractile function in chemotherapy-induced cardiotoxicity (Gripp et al., 2018; Karlsen et al., 2019; Cocco et al., 2022). Cardiac biomarkers offers high sensitivity and specificity, and provides the added benefit of cost-effectiveness, hence blood samples can be tested for biomarkers at closer intervals (Shams-Vahdati et al., 2014). Based on the current limitations in achieving consensus on reliable prognostic tools for early screening of trastuzumab induced cardiotoxicity, the present study identifies a novel panel of biomarkers and circulating plasma miRNAs, which have the potential of providing a superior prognostic modality than conventional approaches. In the present study, patients with breast cancer, receiving trastuzumab therapy, were monitored for progressive cardiac function decline at several time intervals, including pre-chemotherapy initiation, 3-months and 6-months post-chemotherapy initiation. Our results showed significant upregulation of cardiac troponin I and troponin T, which are well established and highly specific predictive markers of cardiac injury and cardiac function decline. These results were in concordance with our previously published findings that showed upregulation of cardiac troponin I and troponin T in anthracyclines induced cardiotoxicity at 3-months and 6-months post chemotherapy initiation (Kitayama et al., 2017; Simoes et al., 2018). The high prognostic efficacy of cardiac troponins has also been confirmed by multiple studies that have shown utilization of these markers for the early assessment of myocardial injury, cardiac degeneration, and remodeling, becoming an effective translational biomarker for cardiotoxicity in humans (Reagan et al., 2013). Hence, utility of these highly sensitive cardiac troponin markers may be highly effective in predicting early onset of cardiotoxicity.

Our study further sets out the prominence of an important pro-angiogenic biomarker, PIGF, which showed significant upregulation at 3-months and 6-months post-trastuzumab therapy initiation. In tumor cells, the expression of PIGF protein have been shown to undergo an angiogenic switch that promotes tumor vascularization (Chau et al., 2017; Saman et al., 2020). This upregulation of PIGF sustains the survival and metastasis of tumor cells while stimulating cardiac angiogenesis (Chau et al., 2017). The anti-metastatic and anti-angiogenic activity of trastuzumab targets the overexpression of HER2 receptors and vascular endothelial growth factor (VEGF) proteins, including PIGF, thereby inhibiting the cardioprotective effects of the HER neuroregulin-1 (NRG-1) ligand, as well as those induced by the cardiac angiogenesis of PIGF (Han et al., 2017). As a result, breast cancer patients administered chemotherapeutic treatments consisting of trastuzumab may be more inclined to develop cardiotoxic effects. Furthermore, a previous study has also shown upregulation of PIGF in breast cancer patients, undergoing combination therapy of anthracyclines and trastuzumab, followed by sensitivity analysis which showed increased risk of developing cardiotoxicity with an increase in the level of PIGF (Putt et al., 2015). Hence, the upregulation of PIGF, as noted in the present study, may be highly relevant to the pathophysiology of trastuzumab-induced cardiotoxicity and could potentially serve as a prognostic marker for cardiac dysfunction following trastuzumab exposure.

Apart from that, upregulated levels of GDF-15 have been predominately studied in relation to major adverse cardiac incidents such as myocardial infarction or heart failure (Kempf et al., 2007; Wollert et al., 2017). GDF-15, a protein member of TGF-β superfamily, have been shown to be highly elevated as a result of cardiomyocytes secreting these proteins in response to stimuli indicative of oxidative stress, myocardial ischemia, proinflammatory cytokines, lower peripheral blood mononuclear cell (PBMC) telomerase activity and cancer (Adela and Banerjee, 2015; Liu et al., 2021). Since oxidative stress is fundamental to the mechanism through which trastuzumab induces cardiotoxicity, the use of this important biomarker is viable in early screening of CRCD. Our results showed significant upregulation of GDF-15 at 3-months and 6-months post chemotherapy initiation, which was in line with the functional role of this biomarker. The findings in the present study were also in concordance with previous studies that positively correlated elevated levels of GDF-15 with cardiac dysfunction in breast cancer patients (Tromp et al., 2020), receiving chemotherapeutic agents, further supporting the predictive utility of this biomarker. Subsequently, the present study also elucidates the potential role of cMLC1, as a biomarker of trastuzumab induced cardiotoxicity. The component of a multimeric protein complex, myosin, cMLC1 is known to facilitate cardiac muscle contractions (England and Loughna, 2013). The circulating levels of cMLC1 has been shown to be upregulated during the injury or damage of cardiomyocytes, as this induces the myocardium to secrete these proteins into circulation (Stejskal et al., 2005). Our study showed significant upregulation of cMLC1 at 3- and 6-months post trastuzumab initiation, suggestive of myocardial damage and progressive decline in cardiac function. These findings were in concordance with previous studies in murine models, which showed that mice treated with trastuzumab exhibited significant damage to cardiac myofibers, suggesting that the structural changes induced by the cardiotoxic effects of trastuzumab diminishes the contractile potential of the heart (Elzarrad et al., 2013). Furthermore, the study also showed significant decline in the cardiac function, which was evident by the echocardiography assessment of these trastuzumab treated mice. Hence, the findings in the presents study and the evidence from literature advocates the potential use of cMLC1 as a predictive biomarker for trastuzumab-induced cardiotoxicity.

The role of miRNAs, as biomarkers, have been increasingly gaining attention as they regulate the transcription of genes associated with the disease progression and offer high sensitivity in detecting early pathophysiological changes associated with the diseased condition (Cho, 2011). The present study further elucidates the intrinsic role and translational applicability of plasma miRNAs in cardiac remodeling, subsequently providing evidence of their prognostic utility in predicting risks of trastuzumab induced cardiotoxicity. Several studies have shown the important clinical utility of highly sensitive miRNAs, due to their specific roles in cardiac injury, inflammation, fibrosis and apoptosis (Cho, 2011). To this end, our results showed significant upregulation of miR-34a, which is primarily expressed in cardiac tissues, after 6-months of trastuzumab therapy. Several studies have shown pro-apoptotic effects of miR-34a on cardiomyocytes as well as mediator of oxidative stress, subsequently showing a positive correlation of elevated expression of miR-34a with cardiotoxicity (Piegari et al., 2016; Pellegrini et al., 2020). Our previous study also showed upregulation of miR-34a in anthracyclines induced cardiotoxicity (Lakhani et al., 2021). Although the role of miRNA-34a in response to trastuzumab has not yet been elucidated in detail, the present study supports the prospective utility of this miRNA in monitoring the cardiotoxic effects induced by trastuzumab treatment, prior to cardiac dysfunction. Apart from that, we also assessed the role of miR-21, which is primarily dysregulated in response to ischemic injury of the heart, precipitated by oxidative stress or inflammation (Wu et al., 2015). Studies have shown that the inhibition of miR-21 expression improves interstitial fibrosis and cardiac function, suggestive of its role in cardiac structural remodeling (Surina et al., 2021). The cardiotoxic effects of trastuzumab is most notably manifested through reduced LVEF, which is characterized by myocardial interstitial fibrosis. Hence, suggesting that circulating levels of miR-21 may serve as a potential predictor trastuzumab induced cardiotoxicity. Notably, our results also showed marked upregulation of miR-21 at 3-months and 6-months post trastuzumab initiation. In addition, our results also showed significant upregulation of miR-133 after 6-months of trastuzumab therapy. The clinical utility of miR-133, as prognostic marker of trastuzumab induced cardiotoxicity, is supported by the evidence in the literature that shows expression of this miRNA primarily in muscle tissues (Yu et al., 2014). Furthermore, miR-133 have been shown to participate in cardiac remodeling, specifically causing cardiac fibrosis and hypertrophy, due to its role in cellular proliferation, hypertrophic growth and electrical remodeling, affecting cardiac dysfunction (Xiao et al., 2019). Hence, cardiac changes ascertained by miR-133 support its role as a prognostic marker for cardiotoxicity. The cumulative line of evidence also showed an important role of miR-1 in chemotherapy induced cardiotoxicity. The upregulation of miR-1 has been shown to cause redox imbalance by direct suppression of antioxidant factors in cardiomyocytes, hence promoting oxidant stress as well as subsequent apoptotic activity leading to cardiac damage (Ai et al., 2010; Zhou et al., 2013). Previous studies have also demonstrated the potential role of miR-1 in causing anthracyclines induced cardiotoxicity, offering superior modality than cardiac troponin I (Rigaud et al., 2017). While the role of miR-1 has not been previously explored in trastuzumab induced cardiotoxicity, our results are in concordance with previous observations, suggesting its utility as a viable prognostic marker. Subsequently, our results showed upregulation of miR-30e in trastuzumab administered breast cancer subjects. Studies have shown that miR-30e has a functional role as a pro-apoptotic factor and promotes autophagy (Zheng et al., 2018). The increased expression of miR-30e and subsequent excessive autophagy results in the cardiomyocyte death during myocardial injury (Li et al., 2018).

Together, the present study demonstrates strong translational utility of the proposed biomarker panel in predicting early onset of trastuzumab induced cardiotoxicity in patients with breast cancer. Despite the strong statistical outcomes of the present study, there were several limitations of the study. The viability of the proposed panel can be further strengthened by confirming the present findings in a large population prior to implementation in a clinical practice. As taxane itself has cardiotoxicity properties, the initial combination therapy with taxane may interfere with the cardiotoxicity induced by trastuzumab therapy. Furthermore, another limitation of the study was the short follow up period of up to 6-months only, since cardiotoxicity can become apparent as the treatment progresses, hence, also affect the level of the proposed biomarkers. Despite the small sample size and shorter follow up period, our results showed apparent cardiotoxicity in about 18% of the patients after only 6-months of trastuzumab therapy. However, the present study still offers crucial evidence and a cost effective, non-invasive predictive modality demonstrating the efficacy of the proposed panel of biomarkers. With advances in the understanding the mechanisms operant in trastuzumab induced cardiotoxicity, more biomarkers may be added to this panel that may be highly specific to the molecular changes in cardiac tissues, induced by trastuzumab therapy. Furthermore, the utilization of this panel in patients with highest risk of cardiotoxicity, due to comorbidities, may allow to monitor cardiotoxic manifestation by trastuzumab therapy. The proposed panel offers a viable guide to the clinicians in developing mitigation strategies, including dose adjustments, mitigation of cardiovascular risks, or alternate treatment therapies. These mitigation strategies can be tailored based on the cumulative evidence including, susceptibility of the patients to develop cardiotoxicity, prior risk factors, presence of other acute or chronic conditions, and finally levels of these biomarkers. The panel will help monitor high risk group for cardiotoxicity, which may need more surveillance during therapy. Nevertheless, the implementation of this panel of biomarkers may improve health outcomes and reduce mortality associated with chemotherapy induced cardiotoxicity.

## Data Availability

The original contributions presented in the study are included in the article/supplementary material, further inquiries can be directed to the corresponding author.
